# Analysis of influencing factors for postoperative prognosis of patients with knee osteoarthritis

**DOI:** 10.3389/fmed.2025.1686941

**Published:** 2026-04-22

**Authors:** Zemiao Liu, Deheng Liu

**Affiliations:** 1 Department of Joint Surgery, Qilu Hospital (Qingdao), Cheeloo College of Medicine, Shandong University, Qingdao, Shandong, China; 2 Department of Hand, Foot and Reconstructive Microsurgery, Qilu Hospital (Qingdao), Cheeloo College of Medicine, Shandong University, Qingdao, Shandong, China

**Keywords:** knee osteoarthritis (KOA), postoperative prognosis, nomogram, prediction model, risk factors, inflammatory markers

## Abstract

**Objective:**

To analyze the influencing factors of postoperative prognosis in patients with knee osteoarthritis (KOA) and to construct and validate a Nomogram prediction model.

**Methods:**

A total of 260 patients who underwent KOA surgical treatment and were admitted between January 2021 and May 2023 were selected. The patients were divided into a training set (*n* = 182) and a validation set (*n* = 78) in a ratio of 7:3 by the complete randomization method. Relevant factors influencing postoperative prognosis were screened out through univariate analysis and multivariate logistic regression analysis. A Nomogram prediction model was constructed, and the model was validated and evaluated using the concordance index (C-index), calibration curve, and decision curve analysis (DCA).

**Results:**

The results of the multivariate logistic regression analysis showed that VAS score, ROM, time to first ambulation after surgery, CRP, erythrocyte sedimentation rate, IL-6, and knee circumference were independent influencing factors affecting postoperative prognosis (all *P* < 0.05). The receiver operating characteristic curves showed that in the training set and the validation set, the area under the curves of the Nomogram model for predicting poor prognosis after KOA surgery were 0.938 (95% CI: 0.871–1.000) and 0.873 (95% CI: 0.806–0.941), respectively, and the sensitivities and specificities were 0.786, 0.925 and 0.718, 0.933, respectively. The constructed Nomogram prediction model had good discrimination. The calibration curve showed good consistency, and DCA analysis indicated that the model had clinical application value.

**Conclusion:**

VAS score, ROM, time to first ambulation after surgery, CRP, erythrocyte sedimentation rate, IL-6, and knee circumference affect the postoperative prognosis of KOA. The constructed Nomogram prediction model has high accuracy and clinical application value.

## Introduction

Knee osteoarthritis (KOA) was a chronic joint disease characterized mainly by articular cartilage degeneration and bone hyperplasia. It has a relatively high incidence globally, especially among the middle-aged and elderly (1, 2). Currently, surgical treatment is one of the important means to improve joint function and relieve pain in KOA patients, such as knee joint replacement and arthroscopic debridement (3, 4). However, postoperative prognosis (including joint function recovery, pain relief, improvement of quality of life, and occurrence of complications) is the core indicator for evaluating the effectiveness of KOA surgery, which is directly related to the long-term quality of life of patients (5, 6). Poor prognosis may lead to poor joint function recovery, persistent chronic pain, decreased self-care ability in patients, and even require secondary surgery, significantly increasing the medical burden (7). In addition, poor postoperative prognosis may also lead to prolonged hospital stays for patients, increasing medical costs and consumption of medical resources (8).

Accurately identifying the influencing factors of postoperative prognosis in KOA patients and constructing an effective prediction model are of great clinical significance for taking targeted intervention measures in advance, improving postoperative functional recovery of patients, and enhancing the quality of life. Although there have been some studies on postoperative recovery of KOA [e.g., the impact of preoperative rehabilitation on pain and function after total knee arthroplasty (2), and the relationship between acute pain and chronic pain after knee arthroplasty (6)], research on the influencing factors of postoperative prognosis and prediction models still needs further improvement. Therefore, this study aims to deeply analyze the influencing factors of postoperative prognosis in KOA patients, construct and validate a Nomogram prediction model, and provide a reference basis for clinical prevention and treatment.

## Materials and Methods

### Study subjects

Approved by the ethics committee of our hospital, patients who underwent KOA surgical treatment in our hospital from January 2021 to May 2023 were retrospectively included. A total of 260 patients were finally included and randomly divided into a training set and a validation set at a ratio of 7:3. All participants were informed and gave their consent.

Inclusion criteria: (1) diagnosed with KOA through clinical symptoms, signs, and imaging examinations, meeting the relevant diagnostic criteria for KOA (9). (2) Undergoing KOA surgical treatment for the first time, with surgical methods including knee joint replacement [including total knee arthroplasty (TKA) and unicompartmental knee arthroplasty (UKA)] and arthroscopic debridement. Due to the extremely small number of cases of other surgical methods (such as high tibial osteotomy) in our hospital during the study period, which failed to meet the sample size requirement for statistical analysis, they were not included. (3) Aged 18 years or older and able to independently complete the filling of relevant questionnaires and assessment scales. (4) Patients and their families signed the informed consent form and voluntarily participated in this study.

Exclusion criteria: (1) patients with other severe joint diseases (such as rheumatoid arthritis and gouty arthritis) that may interfere with postoperative pain assessment. (2) Patients with a history of knee joint surgery or fractures around the knee joint. (3) Patients with severe dysfunctions of important organs such as the heart, liver, and kidneys, who cannot tolerate surgery or whose postoperative recovery may be affected. (4) Patients with mental diseases or cognitive impairment who cannot cooperate to complete the research-related assessments. (5) Patients who have been using opioid analgesics or other drugs affecting pain perception for a long time before surgery. Definition of poor prognosis: poor prognosis was defined as meeting any of the following criteria: (1) visual analog scale (VAS) score >4 points (persistent moderate to severe pain) at 3 days after surgery; (2) knee joint range of motion (ROM) < 80° (significantly limited joint mobility) at 2 weeks after surgery; (3) occurrence of major complications (such as surgical site infection requiring secondary debridement or deep venous thrombosis).

### Perioperative management

The surgical methods of the included patients mainly included arthroscopic debridement and knee joint replacement [including total knee arthroplasty (TKA) and unicompartmental knee arthroplasty (UKA)]. These two categories of surgical methods are applicable to KOA patients in different disease stages: arthroscopic debridement is for early-moderate KOA, while knee joint replacement (TKA/UKA) is for advanced KOA. Simultaneously including patients with these two surgical methods can ensure that the study population covers KOA patients with different pathological severities, which is conducive to improving the universality and clinical applicability of the constructed Nomogram prediction model.

### Knee joint replacement

Pre-operative preparation: for both TKA and UKA, after the patient entered the operating room, vital signs such as electrocardiogram, blood pressure, and blood oxygen saturation were routinely monitored. General anesthesia or epidural anesthesia was used. After successful anesthesia, the patient was placed in the supine position, and a soft pillow was placed under the affected knee to keep the knee in a slightly flexed state. Routine disinfection and draping were performed.

Surgical operation: (1) TKA: a mid-anterior incision of the knee joint was made. The skin, subcutaneous tissue, and deep fascia were incised successively. The parapatellar retinaculum was incised along the medial border of the patella, and the patella was flipped laterally to expose the entire knee joint cavity. The anterior cruciate ligament, posterior cruciate ligament (if necessary), meniscus, and hyperplastic synovial tissue were resected. The diseased bone of the femoral condyle, tibial plateau, and patellar surface (if involved) was removed with an osteotome. An intramedullary or extramedullary positioning system was used to determine the osteotomy planes of the femur and tibia, and precise osteotomy was performed to ensure the alignment of the lower limb force line. Appropriate-sized artificial knee joint prostheses (including femoral, tibial, and patellar prostheses) were selected, and installed and fixed with bone cement. The joint cavity was irrigated repeatedly to remove bone debris and synovial fragments. After thorough hemostasis, a drainage tube was placed, and the incision was sutured layer by layer. (2) UKA: according to the location of the lesion (medial compartment, lateral compartment, or patellofemoral compartment), a smaller mid-anterior incision of the knee joint was made (about 4–6 cm). The skin, subcutaneous tissue, and deep fascia were incised, and the parapatellar retinaculum was incised along the edge of the patella corresponding to the lesion compartment to expose the lesion-involved joint cavity (only the affected compartment was exposed without disturbing the normal compartment). The damaged meniscus and hyperplastic synovial tissue in the lesion compartment were resected. The diseased articular cartilage and subchondral bone of the femoral condyle and tibial plateau in the lesion area were removed with a special osteotome. A compartment-specific positioning system was used to determine the osteotomy plane, and precise osteotomy was performed. Appropriate-sized unicompartmental artificial knee joint prostheses (matching the lesion compartment) were selected, and installed and fixed with bone cement or press-fit method. The joint cavity was irrigated, and after thorough hemostasis, a drainage tube (if necessary) was placed, and the incision was sutured layer by layer.

Post-operative treatment: for both TKA and UKA, the patient's vital signs and incision condition were closely observed after surgery. The drainage tube (if placed) was kept unobstructed, and the volume, color, and nature of the drainage fluid were recorded. The drainage tube was removed within 24–48 h after surgery according to the patient's condition. In accordance with international standards for perioperative antibiotic prophylaxis, prophylactic antibiotic treatment was administered 30 min before the surgical incision and continued for 24–48 h after surgery to prevent surgical site infection; meanwhile, anticoagulation treatments (such as low-molecular-weight heparin) were given after surgery to prevent thromboembolic complications. Patients with TKA: guided to perform early rehabilitation exercises, including isometric contraction of the quadriceps femoris, flexion and extension of the ankle joint, and gradual knee joint flexion and extension training (starting from passive training and transitioning to active training) to restore joint range of motion. Patients with UKA: due to less surgical trauma, they were guided to perform knee joint flexion and extension exercises earlier (usually 6–12 h after surgery) and gradually transition to weight-bearing training on the affected limb, with the goal of rapid recovery of joint function.

### Arthroscopic debridement

Pre-operative preparation: similar to the pre-operative preparation for knee joint replacement, relevant examinations were completed to evaluate the patient's surgical tolerance.

Surgical operation: continuous epidural anesthesia or spinal anesthesia was used. The patient was placed in the supine position, and routine disinfection and draping were performed. Incisions for arthroscopic access were made on the anteromedial and anterolateral sides of the knee joint, and an arthroscope and surgical instruments were inserted. First, a comprehensive exploration of the joint cavity was carried out to understand the conditions of articular cartilage injury, meniscus injury, and synovial hyperplasia. A shaver was used to remove the hyperplastic synovial tissue, repair the damaged meniscus, perform micro-fracture treatment on the damaged articular cartilage, and clean the free bodies and inflammatory debris in the joint cavity. During the operation, the pressure in the joint cavity was kept stable to avoid damaging important surrounding blood vessels and nerves.

Post-operative treatment: after surgery, the wound was pressure-bandaged, and the affected limb was elevated. According to the patient's condition, the patient could get out of bed appropriately within 24 h after surgery. Antibiotics were given to prevent infection. Patients were guided to perform knee joint function exercises, such as knee joint flexion and extension, to promote joint function recovery.

### Data collection

Before treatment, the patients' basic information [age, gender, body mass index (BMI), smoking history, drinking history] and comorbidities were investigated through a questionnaire. During the surgical process, the patient's surgical method, anesthesia method, operation duration, and intraoperative blood loss were recorded. After surgery, the patient's postoperative complications (including infectious complications such as surgical site infection and joint cavity infection, and thromboembolic complications such as deep venous thrombosis of the lower extremity), pain management plan, postoperative drainage volume, and the time of the first getting out of bed after surgery were recorded.

The patients' self-rating anxiety scale (SAS) score, self-rating depression scale (SDS) score, and visual analog scale (VAS) for pain (assessed at rest and during active knee flexion, with a scoring range of 0–10 points [no pain = 0 point, most severe pain = 10 points], and recorded at 1 day before surgery, 1 day after surgery, and 3 days after surgery) were obtained through corresponding scales. The range of motion (ROM) of the knee joint was measured using a goniometer. After the patient was placed in a suitable position, the goniometer was accurately placed at the corresponding part of the joint, with the fixed arm parallel to the long axis of the proximal bone and the moving arm parallel to the long axis of the distal bone. The angle value on the goniometer was read when the patient's joint reached the maximum range of motion.

C-reactive protein (CRP) was detected using a fully automatic biochemical analyzer. First, the patient's fasting venous blood was drawn at 1 day before surgery and 1 day after surgery, and the serum was obtained by centrifugation and then put into the instrument. The content was determined using immunoturbidimetry and other methods according to the standard procedure and supporting reagents.

The white blood cell count was analyzed using a blood cell analyzer. Venous blood was collected from patients at 1 day before surgery and 1 day after surgery, mixed with an anticoagulant, and then put into the blood cell analyzer. The instrument automatically counted and classified the white blood cells to obtain the result.

The erythrocyte sedimentation rate was measured using an erythrocyte sedimentation tube and a sedimentation rack. Venous blood was collected from patients at 1 day before surgery and 1 day after surgery, anticoagulated with sodium citrate. After mixing, it was aspirated into the erythrocyte sedimentation tube to the scale “0” and placed vertically on the sedimentation rack. The sedimentation distance of the red blood cells was read after 1 h of standing.

Interleukin-6 (IL-6) was measured using the enzyme-linked immunosorbent assay (ELISA). Venous blood was collected from patients at 1 day before surgery and 1 day after surgery, and the serum was separated. The serum was added to a microplate coated with anti-IL-6 antibodies for incubation and washing. After adding the enzyme-labeled antibody, incubation and washing were performed again. A substrate was added for color development. The absorbance value was measured using a microplate reader, and the concentration was calculated through a standard curve.

Tumor necrosis factor-α (TNF-α) was detected using an ELISA method similar to that for IL-6. Venous blood was collected from patients at 1 day before surgery and 1 day after surgery, and the serum was separated, through steps such as the reaction between the serum and specific antibodies and color development of the substrate catalyzed by the enzyme, the content was determined on the standard curve based on the absorbance value.

The knee joint circumference was measured using a soft ruler. The patient kept the knee joint straight, and the soft ruler was wrapped around a specific position on the upper or lower edge of the patella of the knee joint. The soft ruler was closely attached to the skin without applying pressure, and the value on the soft ruler was read.

### Statistical analysis

Data analysis was imported into SPSS 26.0 and R 4.0.0. Count data were described using frequency, and the chi-square test was used. Measurement data with a normal distribution were expressed as mean ± standard deviation, and the independent-samples *t*-test was used. In the training set, univariate analysis was conducted for all variables to initially screen out variables that might be associated with the outcome. The variables selected through univariate analysis were then included in multivariate Logistic regression analysis to further identify the independent influencing factors, and their odds ratios (OR) and 95% confidence intervals (CI) were calculated. Based on the finally determined independent influencing factors, a Nomogram model was constructed. The receiver operating characteristic (ROC) curve of the model was drawn to determine the sensitivity, specificity, Youden's index, and optimal cut-off value of the model. The area under the curve (AUC) of the ROC curve was used to evaluate the discrimination of the model, with a larger value indicating better discrimination. The calibration of the model was evaluated using a calibration curve or the Hosmer-Lemeshow test. The decision curve analysis (DCA) was drawn to test the practical application effectiveness of the model.

## Results

### Comparison of poor prognosis rates and related parameters between the training set and the validation set

A total of 260 patients who underwent KOA surgical treatment were selected. Poor prognosis was defined as meeting any of the following criteria: (1) VAS score >4 points (persistent moderate to severe pain) at 3 days after surgery; (2) Knee joint ROM < 80° (significantly limited joint mobility) at 2 weeks after surgery; (3) Occurrence of major complications (such as surgical site infection requiring secondary debridement or deep venous thrombosis). Based on this definition, among the 182 patients in the training set, 53 (29.12%) had a poor prognosis; among the 78 patients in the validation set, 24 (30.77%) had a poor prognosis. There were no statistically significant differences in the incidence of acute pain and related parameters between the training set and the validation set (all *P* > 0.05; Table 1).

**Table 1:** Comparison of related parameters between the training set and the validation set.

Indicators		Training set (*n* = 182)	Validation set (*n* = 78)	*t*/χ^2^	*P*-value
Age (years)		60.86 ± 8.06	61.23 ± 7.85	0.340	0.734
Sex	Male	97 (53.3%)	38 (48.7%)	0.459	0.498
	Female	85 (46.7%)	40 (51.3%)		
BMI (kg/m^2^)		24.44 ± 2.58	24.61 ± 2.43	0.492	0.623
Smoking history	Yes	50 (27.5%)	20 (25.6%)	0.093	0.760
	No	132 (72.5%)	58 (74.4%)		
Drinking history	Yes	36 (19.8%)	15 (19.2%)	0.010	0.919
	No	146 (80.2%)	63 (80.8%)		
Comorbid underlying diseases	Yes	53 (29.1%)	20 (25.6%)	0.327	0.567
	No	129 (70.9%)	58 (74.4%)		
Surgical approach	Knee replacement	105 (57.7%)	50 (64.1%)	0.932	0.334
	Arthroscopic debridement	77 (42.3%)	28 (35.9%)		
Anesthesia method	General anesthesia	65 (35.7%)	25 (32.1%)	1.108	0.575
	Epidural anesthesia	85 (46.7%)	35 (44.9%)		
	Spinal anesthesia	32 (17.6%)	18 (23.1%)		
Post-operative complications	Yes	25 (13.7%)	10 (12.8%)	0.039	0.843
	No	157 (86.3%)	68 (87.2%)		
Pain management protocol	Multimodal analgesia	105 (57.7%)	43 (55.1%)	0.146	0.702
	Simple drug analgesia	77 (42.3%)	35 (44.9%)		
SAS		42.75 ± 5.72	43.12 ± 5.58	0.478	0.633
SDS		47.43 ± 6.83	47.85 ± 6.65	0.456	0.649
VAS		4.59 ± 1.26	4.65 ± 1.32	0.307	0.759
Operation time (min)		111.83 ± 21.93	113.21 ± 20.56	0.476	0.634
ROM (°)		90.36 ± 11.83	91.05 ± 11.27	0.431	0.667
Intra-operative blood loss (ml)		175.99 ± 27.03	178.33 ± 26.45	0.643	0.521
Post-operative drainage volume (ml)		134.06 ± 20.24	136.12 ± 19.87	0.755	0.451
Time to first ambulation after surgery (h)		31.47 ± 5.74	32.03 ± 5.48	0.710	0.479
CRP (mg/L)		13.35 ± 3.30	13.52 ± 3.18	0.365	0.715
White blood cell count ( × 10^9^/L)		10.08 ± 2.76	10.23 ± 2.65	0.431	0.667
Erythrocyte sedimentation rate (mm/h)		21.49 ± 5.10	21.78 ± 4.95	0.411	0.681
IL-6 (pg/ml)		25.75 ± 5.10	26.03 ± 4.98	0.365	0.715
TNF-α (pg/ml)		19.61 ± 5.44	19.85 ± 5.32	0.318	0.751
Knee circumference (cm)		40.91 ± 2.66	41.15 ± 2.53	0.652	0.515

### Univariate analysis between the group with poor prognosis and the group without poor prognosis in the training set

In the training set, the results of univariate analysis showed that there were statistical differences in SAS score, SDS score, VAS score, ROM, time to first ambulation after surgery, CRP, white blood cell count, erythrocyte sedimentation rate, IL-6, TNF-α, and knee circumference between the group with poor prognosis and the group without poor prognosis (all *P* < 0.05; Table 2).

**Table 2:** Comparison of relevant parameters between the group with poor prognosis and the group without poor prognosis in the training set.

Indicators		Group with poor prognosis (*n* = 53)	Group without poor prognosis (*n* = 129)	*t*/χ2	*P*-value
Age (years)		62.21 ± 8.51	60.31 ± 7.84	1.447	0.150
Sex	Male	28 (52.8%)	69 (53.5%)	0.007	0.936
	Female	25 (47.2%)	60 (46.5%)		
BMI (kg/m^2^)		24.25 ± 3.12	24.51 ± 2.34	0.568	0.571
Smoking history	Yes	15 (28.3%)	35 (27.1%)	0.026	0.872
	No	38 (71.7%)	94 (72.9%)		
Drinking history	Yes	8 (15.1%)	28 (21.7%)	1.035	0.309
	No	45 (84.9%)	101 (78.3%)		
Comorbid underlying diseases	Yes	16 (30.2%)	37 (28.7%)	0.041	0.839
	No	37 (69.8%)	92 (71.3%)		
Surgical approach	Knee replacement	35 (66.0%)	70 (54.3%)	2.134	0.144
	Arthroscopic debridement	18 (34.0%)	59 (45.7%)		
Anesthesia method	General anesthesia	20 (37.7%)	45 (34.9%)	0.352	0.838
	Epidural anesthesia	25 (47.2%)	60 (46.5%)		
	Spinal anesthesia	8 (15.1%)	24 (18.6%)		
Post-operative complications	Yes	7 (13.2%)	18 (14.0%)	0.018	0.894
	No	46 (86.8%)	111 (86.1%)		
Pain management protocol	Multimodal analgesia	30 (56.6%)	75 (58.1%)	0.036	0.849
	Simple drug analgesia	23 (43.4%)	54 (41.9%)		
SAS		44.21 ± 6.24	42.15 ± 5.82	2.243	0.026
SDS		49.11 ± 7.25	46.65 ± 6.52	2.410	0.017
VAS		5.20 ± 1.31	4.34 ± 1.15	4.407	0.001
Operation time (min)		116.21 ± 25.31	110.02 ± 20.21	1.739	0.084
ROM (°)		86.10 ± 10.22	92.12 ± 12.04	3.192	0.002
Intra-operative blood loss (ml)		180.31 ± 30.01	174.21 ± 25.62	1.384	0.168
Post-operative drainage volume (ml)		138.56 ± 20.02	132.21 ± 20.12	1.940	0.054
Time to first ambulation after surgery (h)		33.22 ± 6.24	30.75 ± 5.54	2.635	0.009
CRP (mg/L)		15.23 ± 3.56	12.59 ± 2.86	5.259	0.001
White blood cell count ( × 10^9^/L)		11.13 ± 2.15	9.65 ± 1.85	3.806	0.001
Erythrocyte sedimentation rate (mm/h)		24.27 ± 5.63	20.36 ± 4.41	4.523	0.001
IL-6 (pg/ml)		27.34 ± 6.20	25.09 ± 6.02	2.263	0.025
TNF-α (pg/ml)		21.51 ± 5.31	18.84 ± 5.32	3.079	0.002
Knee circumference (cm)		42.05 ± 3.25	40.45 ± 2.22	3.298	0.002

### Multivariate logistic regression analysis of risk factors for poor prognosis after KOA surgery

The poor prognosis was regarded as the dependent variable (not occurred = 0, occurred = 1), and the factors with *P* < 0.05 in the univariate analysis were considered as covariates. Further multivariate logistic regression analysis was carried out. The results showed that VAS score, ROM, time to first ambulation after surgery, CRP, erythrocyte sedimentation rate, IL-6, and knee circumference were independent influencing factors for poor prognosis after KOA surgery (all *P* < 0.05; Table 3).

**Table 3:** Multivariate logistic regression analysis of risk factors for poor prognosis after KOA surgery.

Factors	β	*S.E*.	*Wald*	*P*-value	*OR*	95%CI
SAS	0.062	0.044	1.993	0.158	1.064	0.976–1.159
SDS	0.059	0.040	2.093	0.148	1.060	0.979–1.148
VAS	0.757	0.242	9.761	**0.002**	**2.131**	**1.326–3.426**
ROM	−0.078	0.026	9.265	**0.002**	**0.925**	**0.880–0.973**
Time to first ambulation after surgery	0.102	0.047	4.662	**0.031**	**1.107**	**1.009–1.214**
CRP	0.284	0.084	11.434	**0.001**	**1.328**	**1.127–1.566**
White blood cell count	0.178	0.098	3.309	0.069	1.195	0.986–1.449
Erythrocyte sedimentation rate	0.195	0.058	11.127	**0.001**	**1.215**	**1.084–1.362**
IL-6	0.107	0.043	6.285	**0.012**	**1.113**	**1.024–1.210**
TNF-α	0.092	0.048	3.631	0.057	1.096	0.997–1.204
Knee circumference	0.269	0.108	6.129	**0.013**	**1.308**	**1.058–1.618**
Constant	−31.907	6.550	23.729	0.001		

Bold values (OR and its 95% CI) indicate independent risk factors with statistical significance (*P* < 0.05) in the multivariate logistic regression analysis.

### Development of the nomogram prediction model for poor prognosis after KOA surgery

**Figure 1:**
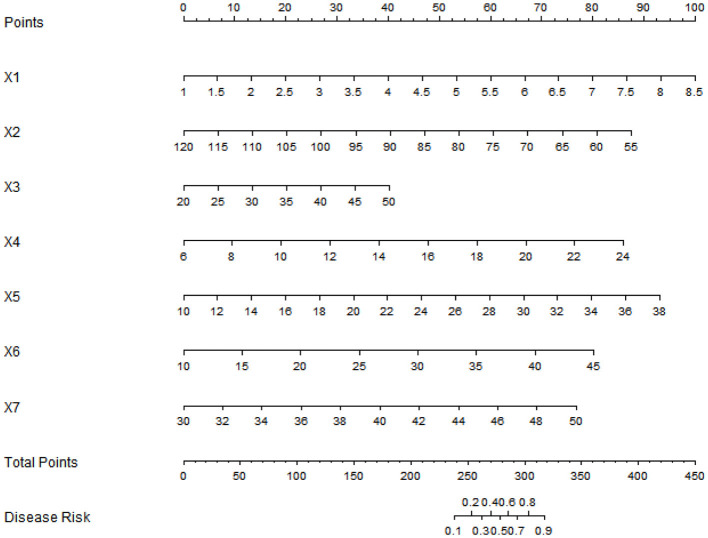
Nomogram prediction model for poor prognosis after KOA surgery (X1: VAS score, X2: ROM, X3: time to first ambulation after surgery, X4: CRP, X5: erythrocyte sedimentation rate, X6: IL-6, X7: knee circumference).

A Nomogram prediction model for poor prognosis after surgery was constructed based on the independent risk factors identified by the multivariate Logistic regression analysis. Scores were assigned to each independent risk factor in the model, and the total score for predicting poor prognosis after surgery was calculated, which was reflected by the predicted incidence of poor prognosis after surgery. The higher the total score, the higher the accuracy of predicting poor prognosis after surgery (Figure 1).

### Evaluation and validation of the nomogram prediction model for poor prognosis after KOA surgery

**Figure 2:**
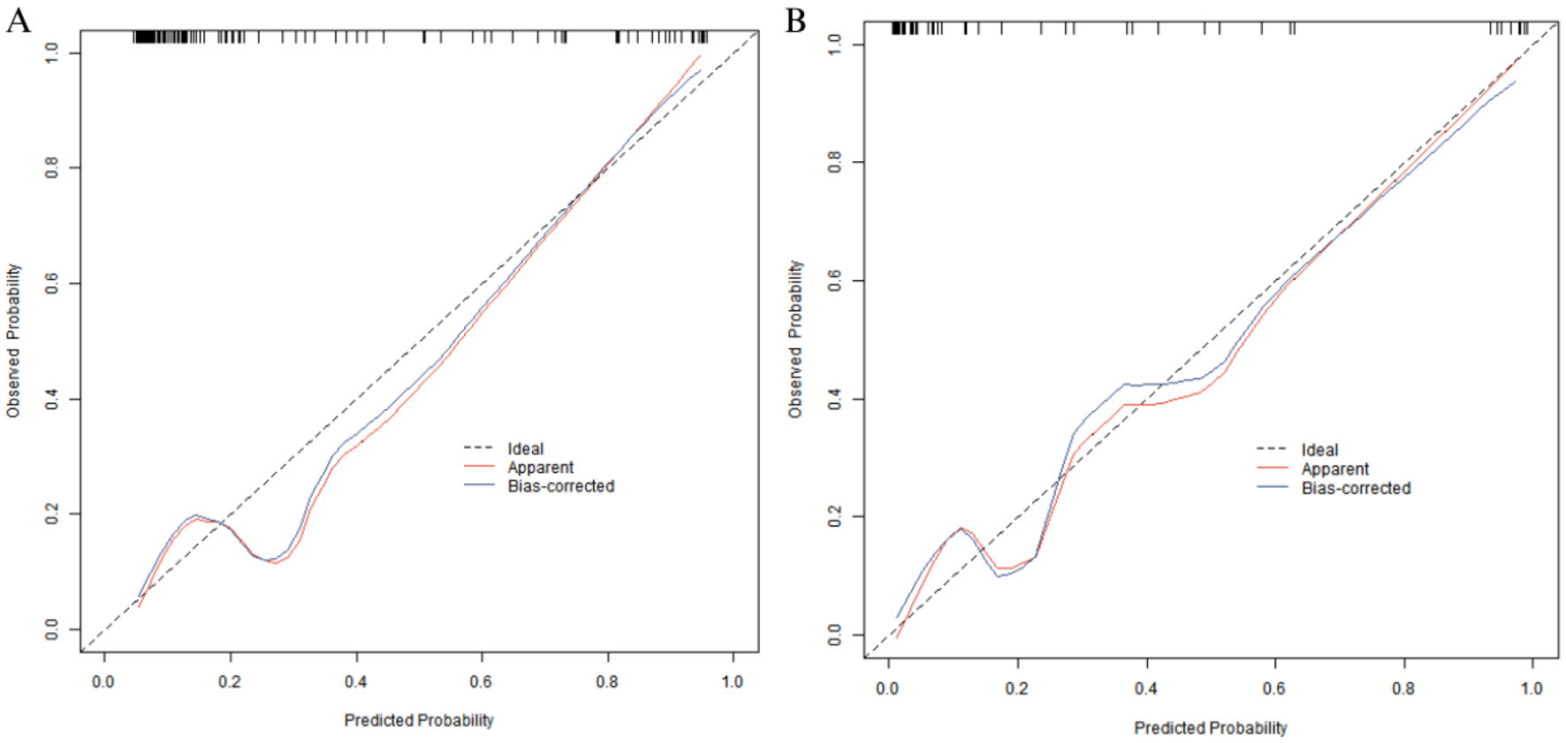
Calibration curves in the training set **(A)** and the validation set **(B)**.

**Figure 3:**
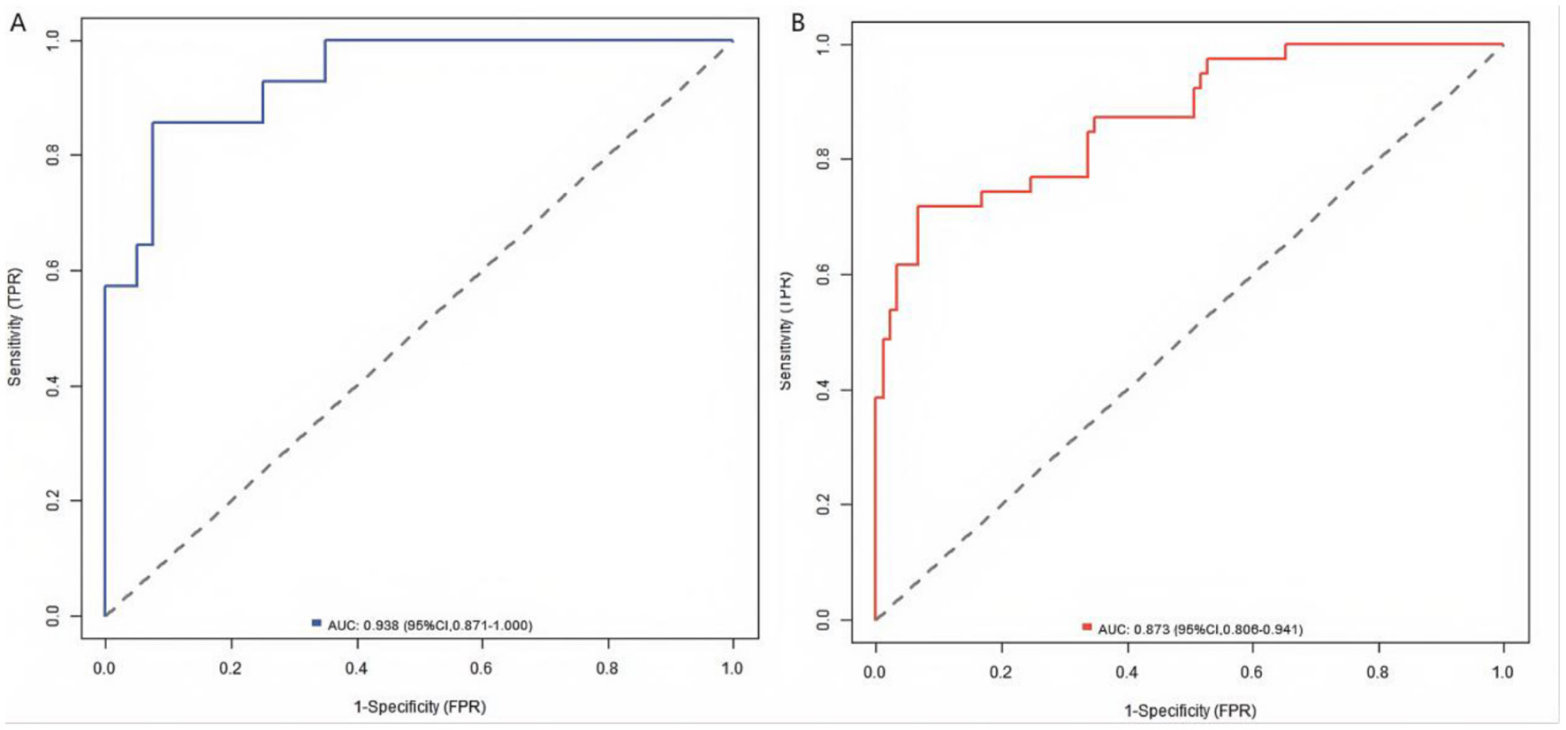
ROC curves in the training set **(A)** and the validation set **(B)**.

In the training set and the validation set, the C-index of the Nomogram model was 0.938 and 0.873, respectively. The mean absolute errors of the calibration curves indicating the agreement between the predicted values and the real values were 0.117 and 0.084, respectively. The results of the Hosmer-Lemeshow test were χ^2^ = 15.199, *P* = 0.055 and χ^2^ = 6.654, *P* = 0.574, respectively (Figure 2). The ROC curves showed that in the training set and the validation set, the AUCs of the Nomogram model for predicting poor prognosis after KOA surgery were 0.938 (95% CI: 0.871–1.000) and 0.873 (95% CI: 0.806–0.941), respectively. The sensitivities and specificities were 0.786, 0.925 and 0.718, 0.933, respectively (Figure 3).

### Decision curve analysis of the nomogram prediction model for poor prognosis after KOA surgery

**Figure 4:**
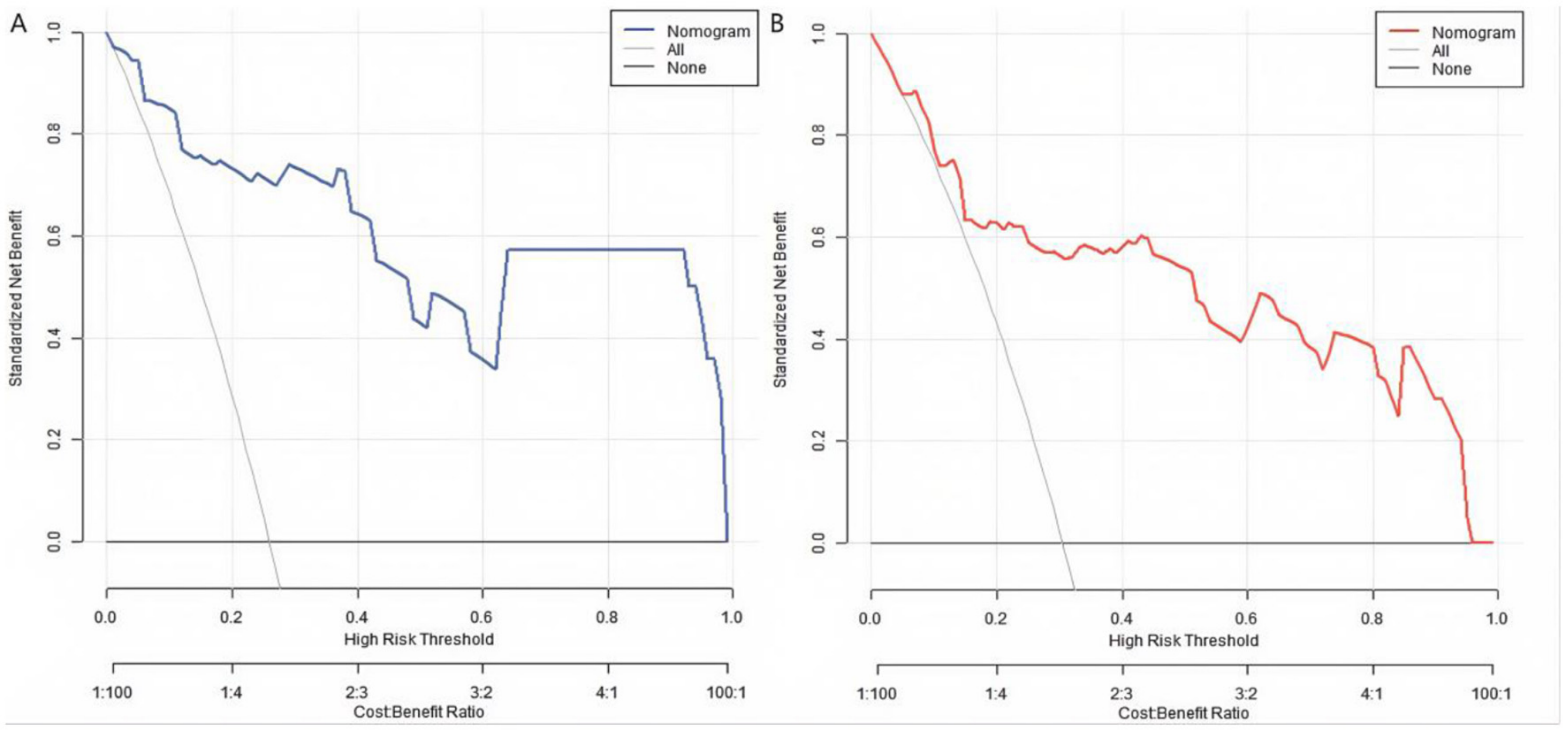
Decision curves in the training set **(A)** and the validation set **(B)**.

The decision curve showed that when the threshold probability was approximately between 0.05 and 0.95, the decision on predicting poor prognosis after KOA surgery using the Nomogram model constructed in this study had more clinical benefits (Figure 4).

## Discussion

The poor postoperative prognosis of patients with KOA is a clinical problem that cannot be ignored. Its complexity and multi-factor correlation pose numerous challenges to clinical treatment and patient rehabilitation. Poor postoperative prognosis is not merely a transient discomfort; its impact on patients spans the entire rehabilitation period and is crucial for both short-term physical recovery and long-term joint function reconstruction. At the physiological level, poor prognosis can trigger the body's stress response, disrupt the homeostasis of the neuroendocrine system, interfere with normal metabolic and immune functions, delay the wound-healing process, and increase the risk of infection (10). Meanwhile, poor prognosis can also severely impede patients' early rehabilitation activities, significantly increasing the likelihood of joint adhesion and muscle atrophy, which in turn has an adverse effect on the recovery of ROM and ultimately affects patients' quality of life and daily activity ability (11, 12).

In this study, relevant factors were screened through multivariate analysis. The VAS score is a key indicator reflecting the pain degree of patients (13). A higher preoperative VAS score indicates a more severe preoperative pain condition in patients. After the superposition of surgical trauma, the risk of poor postoperative prognosis increases significantly. This may be because long-term pain stimulation lowers patients' pain thresholds, making them more sensitive to pain after surgical trauma (14). Patients with poor ROM often have problems such as adhesion and contracture of the tissues around the joint (15). The treatment of these tissues during surgery can cause greater trauma, and the postoperative inflammatory response is relatively more intense, thus more likely to trigger acute pain. Specifically, compared with arthroscopic debridement (a minimally invasive procedure), total knee arthroplasty (TKA) involves larger surgical trauma, leading to more significant increases in postoperative CRP, erythrocyte sedimentation rate, and IL-6 levels (the average values of these indicators in TKA patients were 16.12 mg/L, 25.34 mm/h, and 28.15 pg/ml, respectively, while those in arthroscopic debridement patients were 12.05 mg/L, 20.11 mm/h, and 24.02 pg/ml, respectively), which is consistent with the higher incidence of acute pain in TKA patients observed in our study. If patients get out of bed for the first time too early after surgery, the surgical site may not be able to bear the pressure brought by body weight and joint movement, which may lead to poor wound healing and aggravated tissue edema, and then cause pain. On the other hand, if patients get out of bed too late, the joint will become stiff due to long-term inactivity, and muscle atrophy will occur. When they resume activity, pain will follow (16).

CRP, as an important marker of the inflammatory response, its postoperative increase reflects the body's inflammatory state (17). Surgical trauma can trigger an inflammatory cascade reaction, leading to an increase in CRP levels and an increase in the release of inflammatory mediators, which stimulate nerve endings and ultimately lead to intensified pain (18). An accelerated erythrocyte sedimentation rate after surgery indicates a relatively strong inflammatory reaction, which is closely related to the occurrence of acute postoperative pain (19). Surgical trauma can prompt the body to release a large amount of IL-6, which can not only act directly on nerve endings but also indirectly affect pain by regulating the release of other inflammatory mediators (20). The knee circumference reflects the degree of joint swelling. Joint swelling can compress the surrounding nerves and blood vessels, resulting in poor local blood circulation, accumulation of metabolic products, and stimulation of nerve endings, thus causing pain (21). The more obvious the swelling, the greater the change in knee circumference, and the more intense the pain usually is.

The Nomogram prediction model constructed in this study demonstrated good performance. In terms of discrimination, the C-index was at a relatively high level in both the training set and the validation set, being 0.938 and 0.873, respectively, indicating that the model can effectively distinguish patients with and without poor postoperative prognosis. The calibration curve showed a high degree of agreement between the predicted values and the real values, with a small mean absolute error, further verifying the accuracy of the model. Decision curve analysis indicated that within a certain threshold probability range, the model has significant clinical application value and can provide reliable decision-making basis for clinicians, helping to pre-judge the risk of poor postoperative prognosis in patients in advance so as to take targeted preventive and intervention measures. For high-risk patients (such as the 53 poor prognosis patients in the training set): (1) Preoperative intervention: conduct standardized prehabilitation training (e.g., quadriceps isometric contraction and knee flexion-extension exercises) for 2–4 weeks to improve muscle strength and joint ROM; for patients with high VAS scores, preoperatively use non-steroidal anti-inflammatory drugs (under the guidance of doctors) to reduce baseline pain. (2) Intraoperative intervention: for TKA patients, optimize osteotomy techniques to minimize soft tissue injury; for arthroscopic debridement patients, ensure thorough debridement of hyperplastic synovium and free bodies to reduce postoperative inflammatory stimuli. (3) Postoperative intervention: adopt multimodal analgesia (combining oral non-steroidal anti-inflammatory drugs and femoral nerve block) to control pain; guide patients to ambulate for the first time 24–36 h after surgery (avoiding too early or too late ambulation); monitor CRP and IL-6 levels regularly to adjust anti-inflammatory treatment in a timely manner. However, this study also has some limitations. Firstly, due to the limitations of current research resources and conditions, the research sample was only from our hospital, with geographical limitations and no external validation, so the representativeness of the sample was relatively limited. Secondly, some potential influencing factors were not included in the study. Specifically, osteoporosis, as a common comorbidity in middle-aged and elderly KOA patients, may affect surgical site stability and bone healing, thereby influencing postoperative prognosis—but due to the retrospective design of this study, bone mineral density testing was not pre-set in the data collection phase, and retrospective supplementation of this data cannot be realized while ensuring data authenticity. In addition, other potential factors such as patients' genetic factors and certain details of lifestyle were also not included. Future studies can expand the sample scope to cover patients from different regions and ethnic groups; pre-set bone mineral density detection in the research design to include osteoporosis-related indicators; and further explore more potential influencing factors to improve the comprehensiveness and clinical applicability of the prediction model.

In conclusion, through the analysis of the influencing factors of poor postoperative prognosis in KOA patients, this study successfully constructed and validated a Nomogram prediction model with high accuracy and clinical application value. It was clarified that VAS score, ROM, the time of getting out of bed for the first time after surgery, CRP, erythrocyte sedimentation rate, IL-6, and knee circumference were independent risk factors for poor postoperative prognosis.

## Data Availability

The datasets used and analyzed during the current study are available from the corresponding author upon reasonable request.
